# A Role for Lipid Mediators in Acute Myeloid Leukemia

**DOI:** 10.3390/ijms20102425

**Published:** 2019-05-16

**Authors:** Andreas Loew, Thomas Köhnke, Emma Rehbeil, Anne Pietzner, Karsten-H. Weylandt

**Affiliations:** 1 Department of Medicine B, Ruppin General Hospital, Brandenburg Medical School, 16816 Neuruppin, Germany; andreas.loew@mhb-fontane.de (A.L.); emma.rehbeil@mhb-fontane.de (E.R.); anne.pietzner@mhb-fontane.de (A.P.); 2 Department of Internal Medicine III, University of Munich, 81377 Munich, Germany; thomas.koehnke@med.uni-muenchen.de; 3 Medical Department, Campus Virchow Klinikum, Charité-Universitätsmedizin Berlin, 13353 Berlin, Germany

**Keywords:** AML, immune therapy, PGE2, omega-3, omega-6, lipidomics

## Abstract

In spite of therapeutic improvements in the treatment of different hematologic malignancies, the prognosis of acute myeloid leukemia (AML) treated solely with conventional induction and consolidation chemotherapy remains poor, especially in association with high risk chromosomal or molecular aberrations. Recent discoveries describe the complex interaction of immune effector cells, as well as the role of the bone marrow microenvironment in the development, maintenance and progression of AML. Lipids, and in particular omega-3 as well as omega-6 polyunsaturated fatty acids (PUFAs) have been shown to play a vital role as signaling molecules of immune processes in numerous benign and malignant conditions. While the majority of research in cancer has been focused on the role of lipid mediators in solid tumors, some data are showing their involvement also in hematologic malignancies. There is a considerable amount of evidence that AML cells are targetable by innate and adaptive immune mechanisms, paving the way for immune therapy approaches in AML. In this article we review the current data showing the lipid mediator and lipidome patterns in AML and their potential links to immune mechanisms.

## 1. Acute Myeloid Leukemia

Acute myeloid leukemia (AML) is a complex and biological heterogenous disease. Different mutations lead to alterations in the differentiation of hematopoietic stem cells and are responsible for the accumulation of immature leukemic blast cells in the bone marrow and peripheral blood. AML accounts for approximately 20% of all deaths due to hematologic malignancies, while only comprising 12% of all new cases [1].

The relapse rate after conventional induction chemotherapy is high, particularly in association with adverse chromosomal or molecular aberrations. Therapeutic advances in AML in recent years are mainly attributed to progress in hematopoietic stem cell transplantation techniques and advances in supportive care. 

Increasing evidence suggests that AML as well as other malignancies are sustained by a minor subpopulation with self-renewal potential, referred to as “leukemic stem cells” (LSC) [2], which have been shown to be more quiescent than the bulk of leukemic cells [3]. Current treatments utilizing cytotoxic agents aimed at proliferation might therefore not target LSCs adequately, which in turn can survive treatment and ultimately lead to relapse. Gene expression analyses have shown that LSCs have a similar gene expression profile compared to hematopoietic stem cells (HSC) [2] and that a stem cell rich expression signature in AML blasts correlates with worse prognosis [4]. The knowledge concerning biology and pathophysiology of LSCs has drastically improved over the past decades [5]. It has become especially clear that the microenvironment surrounding tumor cells plays a vital role in carcinogenesis, and growing evidence suggests that it also plays a central role in how tumor cells interact with the immune system [6]. 

The concept of the elimination of minimal residual disease by immunotherapy has shown to be successful—as a proof of principle—in allogeneic hematopoietic stem cell transplantation for postremission therapy, leading to long lasting remissions in a significant proportion of AML cases. 

For patients ineligible for transplantation, alternative therapeutic strategies are mandatory. Immunotherapeutic approaches for clearing of evading AML cells from the stem cell niche involve different monoclonal antibodies including check point inhibitors, adoptive transfer of NK and T cells, T-cell engineering, systemic cytokine administration, and vaccinations with different approaches such as peptides, modified leukemic cells, and dendritic cells [7,8,9,10].

In this context, there has been increased interest in research aimed at lipid mediators such as prostaglandins, as well as other lipid species and their associated regulatory networks, as these can be critical components affecting tumor cell biology, tumor microenvironment, and thus immune mechanisms affecting AML biology as well as response to treatment approaches.

In the following sections we aim to highlight aspects in the field of lipid and lipid mediator biology. In this context immune mechanisms affected will be addressed in order to explore potential links to immunotherapy in the context of hematologic malignancies in general and in AML in particular. 

## 2. Lipids and Fatty Acids in Hematologic Malignancies

As reviewed before, lipid species and the lipidome are highly abundant and essential components of human cells and tissues [11]. Many of these lipid species (e.g., eicosanoids, sphingolipids, glycerolipids) were shown to be changed in the context of tumor disease and might serve as markers as well as targets for new treatment approaches in malignant disorders. Particularly in the context of the tumor surrounding microenvironment lipid species could be important—and modifiable—targets in oncology [12].

Beside an increased de novo synthesis of fatty acids that is required for membrane synthesis and therefore for cell growth and proliferation, AML cells might have an increased lipid catabolism. Fatty acid oxidation (FAO) has been recognized as a relevant component of the metabolic switch in cancer cells where FAO is used for ATP production in conditions of metabolic stress [13]. Indeed, recent in vitro studies have shown that distinct genetic changes in AML are associated with enhanced dynamics and metabolism of lipid species in AML cells [14]. 

Data from the late 1970s found altered lipid compositions of AML cells with a decreased total cholesterol and cholesterol-to-phospholipid ratio, and an increased percentage of unsaturated fatty acids when compared to normal mature neutrophils, but these patterns might be shared by normal immature myeloid cells [15].

Recent studies also demonstrated wide-ranging changes in the plasma [16] as well as bone marrow [17] lipidome in patients with AML. Total plasma fatty acids were found to be depressed in plasma from AML patients, with the attenuation of plasma phosphocholines, triglycerides, and cholesterol esters [16]. However, free fatty acids such as arachidonic acid (AA) 20:4 n-6 and the corresponding precursors gamma-linolenic acid 18:3 n-6 and 8,11,14-eicosatrienoic acid 20:3 n-6 were increased, while many prostaglandins such as PGE2 and 15-keto-PGF2α were reduced in these plasma analyses. Interestingly, AA as well as gamma-linolenic acid 18:3 n-6 and 8,11,14-eicosatrienoic acid 20:3 n-6 tended to be increased slightly more in patients with higher blast counts [16]. While only observed in plasma, and in a very heterogeneous patient population, these observations might indicate a role for AA in the malignant phenotype of AML.

## 3. Omega-6 and Omega-3-Polyunsaturated Fatty Acids and Their Derived Lipid Mediators in Inflammation

Of particular importance with regard to immune processes are lipid mediators derived from long-chain polyunsaturated fatty acids (PUFA) and in particular arachidonic acid (AA). The PUFA are grouped according to the position of the first double bound, counting from the first methyl-, or “omega”-group. Two groups of PUFAs are important for human physiology: omega-6-PUFA and omega-3-PUFA. They are termed *essential*, since mammals cannot synthesize them and they have to be ingested with the diet in sufficient amounts. 

Concerning omega-6-PUFAs, arachidonic acid (AA) and linoleic acid (LA) are the main components. Most mammals can synthesize AA from LA through enzymatic conversion by desaturases. The most important omega-3-PUFAs are α-linolenic acid (ALA) 18:3n-3, eicosapentaenoic acid (EPA) 20:5 n-3 and docosahexaenoic acid (DHA) 22:6 n-3.

**Figure 1:**
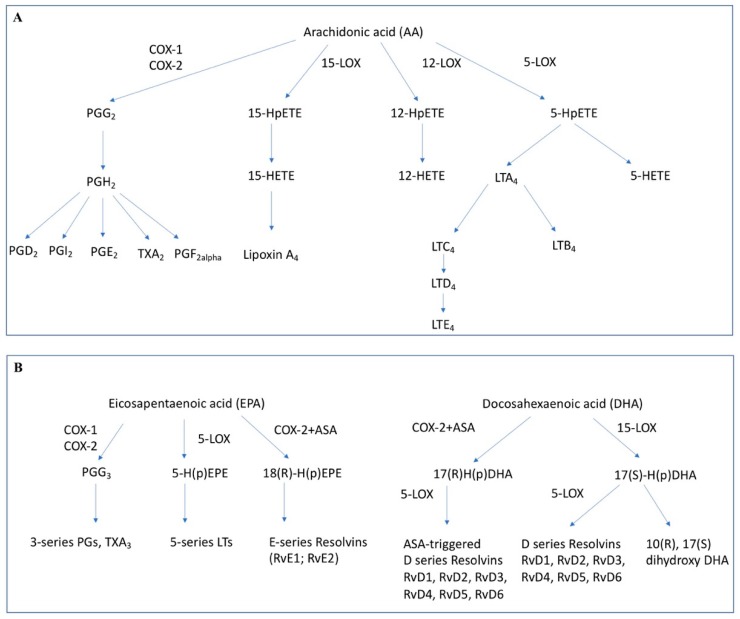
Lipid mediators formed from arachidonic acid (**A**) or eicosapentaenoic acid and docosahexaenoic acid (**B**). COX-1/2: Cyclooxygenase-1/2, 5-LOX: 5-Lipoxygenase, 15-LOX: 15-Lipoxygenase, 12-LOX: 12-Lipoxygenase, ASA: acetylsalicylic acid, PG: prostaglandin, LT: Leukotriene, Rv: Resolvin, HpETE: hydroperoxyeicosatetraenoic acid, HETE: hydroxyeicosatetraenoic acid, H(p)EPE: hydro(pero)xyeicosapentaenoic acid, H(p)DHA: hydro(pero)xydocosahexaenoic acid.

From these PUFA numerous potent lipid mediators are formed (Figure 1). Especially those lipid mediators derived from the omega-6-PUFA arachidonic acid (AA) have been studied intensively. AA is cleaved from its site within phospholipids in the cellular membrane by phospholipase C and A2. Then, AA is further metabolized by two main groups of enzymes: the cyclooxygenases (COX-1 and COX-2) and the lipoxygenases (LOX-5, LOX-12, LOX-15) [18].

The most important enzymes in this pathway are the cyclooxygenases (COX) or prostaglandin endoperoxide H synthases. Two isoforms were identified in the late 1980s and early 1990s. The two cyclooxygenases, COX-1 and COX-2, though derived from different genes of different size, are highly homologous in sequence and three-dimensional structure [19]. They are capable of converting arachidonic acid into prostaglandin (H2), which then is transformed further into prostaglandin E2 (PGE2), amongst others.

PGE2 is the most abundant eicosanoid and has been shown to be a crucial mediator of inflammation, fever, cancer and numerous other physiological systems [20,21,22]. Elevated PGE2 concentrations can be detected in inflamed tissue and the injection of PGE2 causes inflammation [23]. Another lipid mediator derived from AA is thromboxane A2 (TXA2), which is important for platelet function. The effects of PGE2 are mediated through four membrane-bound G-protein coupled receptors—EP1, EP2, EP3, and EP4 [24]. EP1 induces intracellular calcium level variation [25]. EP2 as well as EP4 stimulate cAMP production, which leads to gene regulation. In contrast, EP3 is coupled to Gi and inhibits cAMP production [24]. These receptors differ slightly in their binding characteristics for PGE2 (and to some extent other prostaglandins), as well as their signaling mechanisms, further contributing to a differential biological response due to PGE2 [26,27]. Local amounts of PGE2 are controlled by 15-hydroxyprostaglandin dehydrogenase (15-PGDH) mediated degradation. Overexpression of 15-PGDH can protect from carcinogenesis [28,29] while downregulation of this enzyme can contribute to tumor progression [30,31].

Further groups of lipid mediators derived from AA are the leukotrienes and lipoxins. These are formed by the lipoxygenases. Leukotrienes have pro-inflammatory properties and contribute mainly to allergic reactions, but also play a role in infections and carcinogenesis [18]. Lipoxins are mediators with anti-inflammatory properties [32,33]. Interestingly, it could be shown that under the influence of acetylsalicylic acid (ASA) also the COX enzymes can synthesize potent lipoxins, the so called Aspirin-triggered-lipoxins (ATLs) [32,34].

Omega-3-PUFAs were first postulated to act as anti-inflammatory compounds through the competitive inhibition of PGE2 formation and to a certain degree, EPA and DHA do inhibit the formation of AA derived lipid mediators [35]. Studies have shown increased formation of omega-3-PUFA derived prostaglandins (i.e., PGE3) and decreased formation of AA derived mediators (i.e., PGE2) caused by increased intake of dietary omega-3 PUFA [35,36]. Mechanistically, eicosanoids derived from omega-3-PUFA seem to have a lower biological effect than their omega-6-PUFA derived counterparts [37,38]. However, there is also evidence for some distinct functionality, since PGE3 could be shown to have an inhibitory effect on tumor cell growth in vitro [39]. The same is true for the leukotrienes derived from omega-3-PUFA. For example, for leukotriene B5, which is formed through enzymatic conversion by 5-lipoxygenase. Asthmatic subjects receiving omega-3 supplements showed decreased formation of leukotriene B4 (omega-6-PUFA) and increased formation of leukotriene B5 while displaying improved pulmonary function compared to the control group [40].

In addition to these prostaglandin and leukotriene mediators further omega-3-PUFA derived lipid mediators also play important roles in the course of inflammation. Through enzymatic conversion by lipoxygenases, COX enzymes or cytochrome P450 enzymes, the omega-3-PUFAs DHA and EPA can be converted into potent anti-inflammatory oxylipin mediators [41,42,43]. Particularly the Specialized Proresolving Mediators (SPM) derived from omega-3 PUFA, the resolvins, maresins and protectins were characterized in detail since their initial discovery in 2000 [44] and were found to widely regulate immune cell function [33]. 

Beside the receptors on the cell membrane like EP1-3, transcription factors from cytoplasm and nucleus play an important role in the signaling of inflammatory process and their key mediators. The Peroxisome proliferator-activated receptors (PPARs) are ligand-activated transcription factors belonging to the nuclear receptor family. Their three subtypes, PPARα, PPARβ/δ, and PPARγ have different expression levels in various tissues, biological activity and ligand affinity [45,46]. PPARs are important players in the lipid signaling network between the cell surface and the nucleus. Fatty acids and eicosanoids which also signal through membrane receptors are natural PPAR ligands. For example, PPAR-α is activated by different compounds including arachidonic acid metabolites (LTB4), fibrates and eicosanoids or prostaglandin J2 (15d- PGJ2) is a ligand of PPAR-γ. The activation of PPAR was shown to inhibit the transcription of inflammatory response genes (such as IL-2, IL-6, IL-8, TNF-α) by negatively interfering with the NF-κB, STAT and AP-1 signaling pathways [47]. It is suggested that PPARγ as a transcription factor and its ligands contribute in regulation of a variety of factors related to tumorigenicity [48]. PPARγ could be a target for AML treatment, several ligands with potential anti-leukemic effects have been identified [49]. 

The nuclear factor NF-κB is part of this lipid signaling network. NF-κB influences, as a rapid-acting transcription factor, many processes including immune response and inflammation. Five different proteins (IkBs) inhibit NF-κB in unstimulated cells. NF-κB Proteins are activated through phosphorylation of IkB proteins by the ikB kinase complex, the result is the translocation of NF-κB to the nucleolus. Via TNF- and IL-receptors on the cell surface, proinflammatory cytokines like TNF-α and IL6 activate NF-κB and Stat-3 System. NF-κB itself induces the transcription of TNF-α and with the expression of COX-2 the release of PGE2 [50]. 

The involvement of TNF-α/NF-κB and IL6/Stat3 pathways in tumorigenesis have been confirmed in a series of mouse models of GI malignancy focusing on inflammatory network of the tumor microenvironment [51,52]. 

## 4. Inflammatory Mediators, Immune Function, and Tumor Progression

In the tumor microenvironment, a variety of inflammatory mediators, such as cytokines (IL-6, IL-10, VEGF, TNFα, and TGFβ), chemokines (CCL20 and CXCL8) as well as lipid mediators (such as PGE2) are continuously produced [53]. These mediators are postulated to form a critical interface between immune and neoplastic compartments. Not only do they continuously support tumor survival and expansion, but suppress the function of immune cells, notably, dendritic cells (DCs)—the powerful antigen presenting cells that are crucial for induction of tumor-specific immune responses [53]. In a study from Sombroek et al. examining the supernatants of primary tumor cells (colon, breast, renal cell carcinoma, and melanoma), negative impact on DC development by the factors contained in the supernatants could be demonstrated. Among the factors for which hampering of the differentiation of DCs is known (IL-10, TGF-β1, VEGF, IL-6, M-CSF, and PGE2), only PGE2 was present in such concentrations in the tumor supernatants to show inhibitory effects on the acquisition of DC morphology [54]. Paradoxically, PGE2 also enhances the maturation, migration, and antigen-presenting capacity of DCs. In an effort to explain these seemingly contradictory effects a recent study by Shimabukuro-Vornhagen et al. suggests that whether PGE2-treatment results in inhibition or stimulation of T-cells is dependent on the DC to T-cell ratio during their interaction, showing an inhibitory effect at high DC to T-cell ratios [55]. The authors go on to speculate that this mechanism could serve as a counter-regulatory response in the context of physiologic immune response: Further T-cell activation then would be limited once a large number of mature DCs have accumulated [55].

However, other cell types actively contribute to the immunosuppressive environment within tumors. Myeloid-derived suppressor cells (MDSC) have been found in various cancers. MDSC consist of immature myeloid cells and display a diversity of phenotypes, whereby factors contained in the tumor microenvironment seem to have a major effect on their phenotype and function [56]. They are capable of suppressing adaptive and innate anti-tumor immune responses [57]. PGE2 has emerged as a key molecule in MDSC biology [58]. It not only induces the formation of MDSC (through the EP2 receptor) [59], but also promotes MDSC recruitment to the tumor microenvironment and stabilizes the MDSC phenotype [58].

It has been shown recently that the COX2/PGE2 pathway is involved in the regulation of immune checkpoints by influencing the programmed cell death ligand 1 (PD-L1) expression in tumor-infiltrating bone marrow derived myeloid cells, primarily MDSC and macrophages, and that the inhibition of PGE2 formation is able to attenuate the tumor induced PD-L1 expression [60]. 

Aside from MDSC, regulatory T-cells or Tregs play a role in tumor immune escape. These cells infiltrate the tumor microenvironment and dampen anti-tumor immune responses by inhibiting effector T-cell function [61]. Though the specific mechanisms are yet to be elucidated. Further, Tregs seem to suppress T-cell activity in a PGE2-dependent manner, which can be reversed by COX-2 inhibitors or EP-receptor antagonists [62]. Beside the mediation of suppressive functions COX-2 derived PGE2 from DCs enhances the generation of Tregs and their expansion [63,64]. In peripheral blood of AML patients the frequency of Tregs is significantly higher in comparison to healthy individuals [65]. 

Data from a murine AML model show that PD-1 signaling and regulatory T-cells collaborate to resist the function of cytotoxic T lymphocytes in advanced AML [66]. 

**Figure 2:**
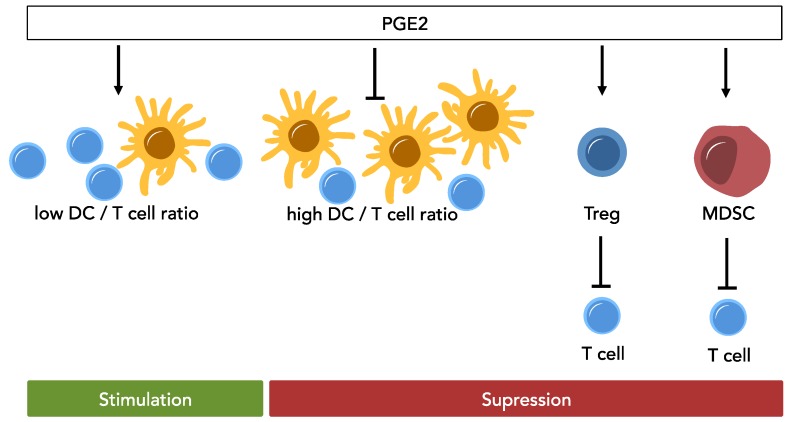
Effect of PGE2 on anti-cancer T cell activity. PGE2 has a differential impact on T cell activity, showing stimulatory effects at low DC/T cell ratios, but suppressive effects as DC numbers increase. PGE2 increases activation of Tregs and is involved in MDSC formation, which in turn hampers anti-cancer immunity. MDSC: myeloid derived suppressor cell; Treg: regulatory T cell; DC: dendritic cell.

One report investigated the role of COX-2 inhibition on indoleamine 2,3-dioxygenase 1 (IDO1) mediated immune dysfunction in AML [67]. IDO1 has been shown to contribute to activation of Tregs, which in turn hamper anti-cancer immunity. In the report by Iachininoto et al., the authors were able to show in vitro that inhibition of the COX-2/PGE2 pathway reduced the expression of IDO1 and inhibits the formation of Tregs [67]. These data, together with the observation that those AML-patients presenting with a high frequency of Tregs at diagnosis were shown to have worse responses to induction chemotherapy, have potential implications to optimize immunotherapeutic approaches [68]. PGE2 thus has a central role in the modulation of immune function as is summarized in Figure 2.

Another approach to modify immunotherapeutic approaches could be based on omega-3 PUFA-derived SPM, which have recently been shown to decrease tumor debris-associated inflammation in an experimental model of tumor debris-stimulated tumor cell proliferation and macrophage-associated inflammation. Compounds such as resolvin D1 (RvD1), RvD2, and RvE1 were able to increase macrophage phagocytosis of tumor cell debris and to decrease the release of cytokines/chemokines from human macrophages stimulated with cell debris [69].

## 5. PUFA-Derived Lipid Mediators in Benign Hematopoiesis

Aside from the presence and effects in terminally differentiated blood cells, the expression and the function of COX isoenzymes and lipid mediators formed by these enzymes in hematopoietic progenitors and precursors remain subject of investigation [70]. Studies in the last decade have provided some insights into the role of the eicosanoid PGE2 in hematopoietic regulation [20]. In particular the stable PGE2-derivative 16,16-dimethyl-PGE2 (dmPGE2) was shown to increase the frequency of long-term repopulating hematopoietic stem cells (HSCs) in irradiated murine bone marrow [71]. This effect was further enhanced by combining dmPGE2 treatment with DPP-4 inhibition using sitagliptin in a mouse model [72].

Furthermore, HSCs pulsed with PGE2 were shown to display a higher (short term) competitiveness, as determined by a head-to-head comparison in a murine competitive transplantation model [20,21,73]. In the context of HSCT, trafficking of HSCs from the peripheral blood to bone marrow niches in the recipient patient, i.e., HSC homing, has been shown to increase under the influence of PGE2 [20]. 

Improving engraftment is especially relevant in the context of umbilical cord blood (UCB) transplantation. UCB transplantations offer some advantages over other sources of HSC, such as lower immune-matching requirements and to some degree a higher availability as UCB is cryopreserved [74]. However, the main pitfall of UCB transplantation is less efficient engraftment than in HSCT from other sources. Utilizing dmPGE2-treatment, Cutler et al. could show promising results in a phase I study by ex vivo-pulsing of UCB with dmPGE2 [75]. Furthermore, also inhibition of 15-PGDH, and thus increase of local PGE2 concentration can contribute to bone marrow transplant recovery [76].

Within the bone marrow, PGE2 is secreted by osteoblasts in large amounts, and given their close physical proximity to HSCs in the bone marrow niche, PGE2 is available to HSCs for the paracrine regulation of stem and progenitor function [20]. 

**Figure 3:**
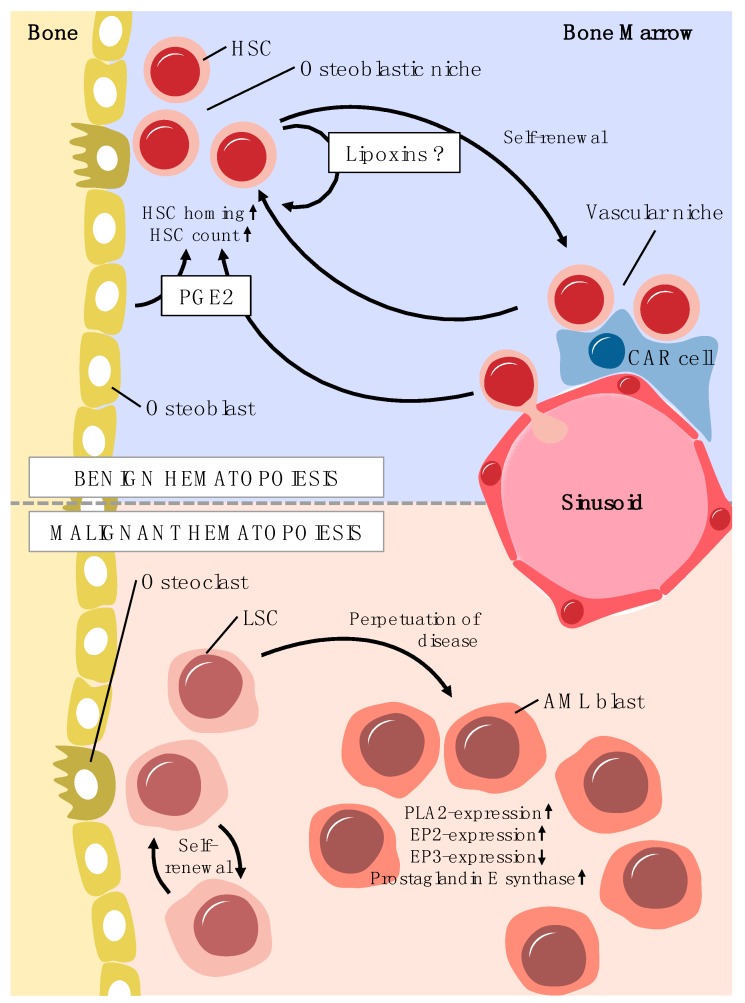
Lipid mediators in benign and malignant hematopoiesis. In benign hematopoiesis, PGE2 is secreted in large amounts in the osetoblastic niche and increases stem cell homing, and long-term LSC numbers. The role of lipoxins is still not fully understood, however lipoxins are required for stem cell quiescence and long-term renewal. In AML, LSC are considered to be chemoresistant and responsible for disease relapse. Self-renewal and maintenance of LSC in the bone marrow niche are increasingly better understood and growing data show alterations of lipid pathway enzymes suggesting eicosanoid pathways are active in leukemic blasts. HSC: hematopoietic stem cell, LSC: Leukemic stem cell; CAR cell: CXCL12-abundant reticular cell; PGE2: Prostaglandin E2; EP2: Prostaglandin E receptor 2; EP3: Prostaglandin E receptor 3; PLA2: Phospholipase A2.

Historically, however, there has been conflicting data on whether PGE2 stimulates or inhibits the growth of hematopoietic progenitor cells. Older studies demonstrated an inhibitory effect of PGE2 on mouse and human myeloid progenitor cells in vitro [77,78]. Further studies revealed that dose, timing, and duration of PGE2-exposure are critical for positive or negative effects on proliferation. Since PGE2 is also produced by the hematopoietic cells themselves, it is therefore postulated that PGE2 might act as a feedback regulator of myelopoiesis [20]. Together, these data suggest that in benign hematopoiesis, PGE2 plays a central role in the HSC niche (Figure 3).

In addition to these data, recent studies in zebrafish and mice have identified the arachidonic-acid derived cytochrome P 450 metabolite 11,12-eipoxyeicosatrienoic acid (11,12-EET) as potent factor to increase embryonic hematopoiesis and adult marrow engraftment [79,80].

## 6. PUFA-Derived Lipid Mediators in Malignant Hematopoiesis

While omega-3 PUFA have been widely implicated in anti-tumor effects in a variety of solid tumors, albeit with mixed results [81], data in hematological malignancies are sparse. In a review published by Betiati et al., a Scopus and PubMed database search between 1998 and 2012 returned 6 studies published on the subject of the effect of omega-3-PUFAs on hematological malignancies [82]. Since then, only few publications regarding effects of omega-3 PUFAs in hematological malignancies have been published. There is some evidence showing a lower incidence of non-Hodgkin lymphomas (NHLs) in patients on a diet high in omega-3-PUFAs [83]. Another study established higher omega-3-PUFAs in NHL patients in remission as compared to those with active disease [84] and recent data implicate low plasma omega-3-PUFAs as marker of inferior prognosis in diffuse large B-cell lymphoma [85]. However, no reduced risk with higher omega-3 PUFAs for lymphoid and myeloid leukemia could be detected so far [86]. In the context of ALL omega-3-PUFAs were shown to be able to lower treatment-related hypertriglyceridemia [87]. Noteworthy is a small recent study with a total of 22 leukemia or lymphoma patients in which a prolonged overall survival time of patients receiving fish oil (2g/d) was shown. These observations might indicate that EPA and DHA improve the response to treatment with conventional chemotherapy in hematological malignancies [88]. In vitro data show an inhibition of cell growth in AML cell lines by EPA and DHA [89,90]. In the erythrocytes of multiple myeloma (MM) patients, Jurczyszyn et al. demonstrated a decreased n-3/n-6 ratio and lower levels of EPA, despite higher levels of its precursor, α-linolenic acid, were measured. This might suggest an impaired functionality of desaturase and elongase enzymes in these patients [91]. A recent systematic review has assessed the current knowledge regarding the omega-3 PUFA EPA and DHA in the context of cells and models of malignant hematopoiesis [92]. There is a wealth of data gained in different cell models, but generally accepted mechanisms, and applicability in vivo and in humans, are still uncertain.

In contrast to this rather limited experimental evidence regarding omega-3 PUFA in the context of malignant hematopoiesis, the main focus of research in this field has been the omega-6 PUFA derived PGE2. Transcript levels of soluble phospholipase A2 (PLA2) subtypes IB and X have been shown to be upregulated in AML blasts compared to control blood mononuclear cells [93]. This finding might be significant since the enzymatic activity of PLA2 releases the eicosanoid precursor, arachidonic acid (AA) from membrane phospholipids for the generation of COX- and lipoxygenase-derived lipid mediators and is in keeping with the higher levels of free AA observed recently in plasma from AML patients [94].

PGE2 might normally act as a feedback regulator of myelopoiesis as described above [20]. However leukemic cells were shown to be resistant to this feedback mechanism seen in benign hematopoiesis [77,95,96]. Furthermore, insensitivity to PGE2-mediated growth inhibition in marrow cultures from patients with myelodysplastic syndrome preceded the patient’s progression to acute leukemia [97]. In keeping with these results, the overall incidence of hematologic malignancies seems not to be reduced by long-term intake of the COX-inhibitor acetylsalicylic acid [98].

In contrast, treatment of mice with indomethacin (a COX inhibitor and thus inhibiting PGE2 synthesis) prior to injection of erythroleukemia cells significantly reduced the number of leukemic cells in both spleen as well as bone marrow [99]. Additionally, Wang et al. could demonstrate a marked reduction of leukemia-initiating cells in a murine limiting dilution transplant assay after treatment with indomethacin [100]. These data indicate a role for COX-metabolites in the proliferation of leukemic cells. 

While COX-1 and COX-2 transcripts can be detected by polymerase chain reaction, the COX-2 protein is not present in primary AML and ALL blasts which is concordance with in vitro data from the human promyelocytic leukemia cell line HL-60 [70,101]. However, AML blasts were shown to express COX-1 [101]. Interestingly, constitutive expression of COX-1 can be upregulated by tumor necrosis factor-related apoptosis-inducing ligand (TRAIL) in HL-60 cells. This is accompanied by an increase of PGE2 synthesis and shows a protective effect towards TRAIL-induced apoptosis [70]. Similarly, AML cells treated with doxorubicin showed overexpression of multidrug transporter MDR1 triggered by increased PGE2-formation, and thereby decreased cytostatic efficacy of doxorubicin [102]. These studies suggest that protective mechanisms of the leukemic blasts to avoid eradication are—at least in part—mediated by PGE2.

In vitro data with AML-mesenchymal stroma cells (AML-MSC) co-cultures show a greatly increased COX-2 expression in MSC and induced PGE2 production in dependence of IL1β and ARC (apoptosis repressor with caspase recruitment domain). ARC is a protein that regulates leukemia microenvironment interactions through NFκB/IL1β and was shown to be an adverse prognostic marker in AML [103]. The COX-2 derived elevation of PGE2 from stromal cells seem to support AML chemoresistance through the expression of β-catenin which regulates ARC [104]. These data indicate that PGE2 production in the microenvironment takes part in a mechanism of an antiapoptotic action and microenvironment-mediated chemoresistance in certain subgroups of AML. 

Downstream of the COX enzymes the last step in the synthesis of PGE2 is performed by the prostaglandin E synthase. This enzyme was shown to be present in normal tissues in minor amounts but is strongly upregulated in neoplastic cells [105,106]. In AML cell lines the prostaglandin E synthase was shown to be upregulated and specific inhibition of the enzyme resulted in an inhibition of proliferation [107] (Figure 2).

As shown for solid tumors, the inhibition of PGE2 receptors EP1, EP2, and EP4 allows for inhibition of cancer-associated inflammation and tumor growth. Mice deficient of these PGE2 receptors display decreased tumorigenesis as demonstrated in various experimental settings mainly for solid tumors [57,108,109,110]. Concordantly, expression levels of EP1 and EP2 have been demonstrated to be increased in cancerous tissues [111]. In AML, Ross et al. as well as Yagi et al. could demonstrate elevated transcript levels of EP2 in AML blasts in a pediatric cohort [112,113] and Denizot et al. could show that AML blasts express functional EP2 receptors [25,114] (Figure 2).

Interestingly, an omega-3 PUFA-derived lipid mediator has been implicated in anti-leukemia effects: The cyclopentenone prostaglandin Δ12-PGJ3, produced through cyclooxygenase action from the omega-3 PUFA EPA, was able to decrease leukemia burden in two murine models of leukemia [115] by selectively targeting leukemia stem cells (LSCs).

Concerning other eicosanoids and their role in hematopoiesis, only limited data has been published. For instance, lipoxins, which are produced by the lipoxygenases (as well as by the COX enzymes if acetylated by ASA), were shown to suppress tumor angiogenesis [116]. Actually, defective lipoxin synthesis was found in leukemia [117], indicating a stabilizing role for lipoxin in benign hematopoiesis (Figure 2). Additionally, a landmark study by Kode et al. demonstrated that an activating β-catenin mutation in osteoblasts can induce the development of leukemia by activating Notch signaling in hematopoietic precursors [118]. Here, osteoblasts exhibited increased expression of the Notch ligand Jagged 1. Conversely, there is some evidence suggesting that lipoxin A4 might decrease the expression of Jagged 1 [119]. Generally, however, the relevance of the lipoxygenase-pathway in benign as well as malignant hematopoiesis is yet to be explored further.

Concerning 5-lipoxygenase, Gal et al. could show elevated transcript levels of 5-lipoxygenase in the CD34+/CD38+ fraction of AML blasts compared to the less mature CD34+/CD38- fraction of the same patients [2]. In a model utilizing mice deficient in 12/15-lipoxygenase it was found that this enzyme is required for the maintenance of long-term HSC quiescence as well as self-renewal [120]. 

## 7. Conclusions

Recent treatment approaches in AML focus increasingly on immune therapy. One of the challenges in the field is to eliminate or reprogram the immune suppressive microenvironment often created by tumors [56]. Interestingly, the otherwise pro-inflammatory lipid mediator PGE2 seems to play a major role in mediating some of these suppressive effects by either direct inhibition of effector T-cell function or indirectly by increasing the frequency of immunosuppressive cell types. Particularly approaches to lower PGE2 might thus serve to enhance immune therapy approaches Current experimental data confirm the importance of this aspect also in the context of AML.
